# Health and social impacts of California wildfires and the deficiencies in current recovery resources: An exploratory qualitative study of systems-level issues

**DOI:** 10.1371/journal.pone.0248617

**Published:** 2021-03-26

**Authors:** Annie Rosenthal, Eric Stover, Rohini J. Haar

**Affiliations:** 1 University of California, Hastings College of the Law, San Francisco, California, United States of America; 2 Human Rights Center, University of California School of Law, Berkeley, California, United States of America; 3 Division of Epidemiology, School of Public Health, University of California, Berkeley, California, United States of America; University of California, UNITED STATES

## Abstract

**Background:**

Wildfires in California have become more deadly and destructive in recent years, and four of the ten most destructive fires occurred in 2017 and 2018. Through interviews with service providers, this article explores how these recent wildfires have impacted surrounding communities and the role various recovery resources have played in responding to the short- and long-term health and social needs of survivors.

**Methods:**

Using a purposive sampling methodology, we interviewed 21 health and social service personnel who assisted in wildfire recovery efforts in California in 2017 and 2018. The study participants worked or volunteered in medical facilities, social services agencies and philanthropy/nonprofit organizations located in communities affected by wildfires. Participants were asked about three common, overarching themes that fire-impacted communities navigate post-disaster: health issues, social issues, and response and recovery resources. Inductive coding was used to identify common subthemes.

**Results:**

The two most frequently discussed social issues during interviews were housing and employment access. Mental and emotional well-being and access to health resources were identified as being the most challenging health concerns that survivors face post-disaster. Participants also identified the following private and public recovery resources that survivors use to attempt to restabilize following the fire: community support, county agencies, the Federal Emergency Management Agency (FEMA,) insurance companies and philanthropic organizations. However, participants noted that the cumulative impacts of these efforts still leave many of their patients and clients without the resources needed to restabilize emotionally, financially and physically. Finally, participants spoke about the community-wide, downstream impacts of wildfires, noting that “survivors” are not only those whose health is immediately compromised by the disaster.

**Conclusion:**

Given the worsening wildfire seasons in California, we must increase our understanding of both the scope of the health and social issues that survivors navigate following a disaster, as well as the effectiveness and sustainability of recovery resources available to survivors. We must also understand the “ripple effect” that wildfires have on surrounding communalities, impacting housing access, social services, and health care access. More research and support, especially during the current COVID-19 pandemic, is urgently needed to improve our ability to support the health and social needs of wildfire survivors in the future.

## Introduction

The Federal Emergency Management Agency (FEMA) defines wildfire as “an unplanned, unwanted fire burning in a natural area, such as forest, grassland or prairie” [1]. While wildfires are, in some respects, essential to the health of California’s ecosystem and have historically caused only modest threat to humans, the scale, frequency and destructiveness of California’s wildfires are growing dramatically and dangerously [2]. Between 2015 and 2020, the state saw seven of its ten most destructive wildfires with four of these fires occurring in 2017 and 2018 alone. This includes the most destructive and deadly wildfire in the state’s history—the Camp Fire in Butte County which burned over 18,000 structures, killed 85 people and burned 153,336 acres in November 2018 (see Table 1) [3,4]. In 2020 more than 9,000 wildfires have burned over 4 million acres (more than 4% of the state’s land,) causing displacement, poor air quality and in some regions, orange tinted skies [5,6].

**Table 1:** 2017 & 2018 California wildfires.

Fire	Location	Date Started	Acres Burned	Structures destroyed	Fatalities
Camp	Butte County	November 2018	153,336	18,804	85
Woolsey	Los Angeles & Ventura	November 2018	96,949	1,643	3
North Bay (Atlas, Tubbs & Nuns)	Napa, Lake, Mendocino & Solano	October 2017	~ 245,000	8,900	44
Thomas	Ventura & Santa Barbara	December 2017	281,893	1,063	2 (plus 23 from the Montecito mudslide)
North Bay Fires (Atlas, Nuns &Tubbs)	---	---	---	---	---
Atlas	Napa & Solano	October 2017	51,642	783	6
Nuns	Sonoma	October 2017	54,382	1,355	3
Tubbs	Napa & Sonoma	October 2017	36,807	5,636	22

Source: [3,7,8].

There are a number of reasons why wildfires have become increasingly deadly and destructive in recent years, including the expansion of the wildland-urban interface (WUI)—the area where homes are located within or adjacent to wildland vegetation. Between 1990 and 2010, the United States saw a 41% growth of the WUI, from 30.8 million homes in 1990 to over 43 million homes in 2010. Given that the majority of fires are started by human activity, the expansion of the WUI is correlated not only with more destructive wildfires, but with more fire ignition [9]. Long-standing fire suppression practices in the state have also added to the increasingly devastating wildfire seasons, as these practices have led to a build-up of vegetation which creates dangerous wildfire fuel [10]. Both of these risk factors are greatly exacerbated by climate change, which has increased the length of the annual fire season and created hotter and drier conditions across the state [11]. The climatic changes intensifying California wildfires are projected to worsen in coming years [12], making it critical to understand the full scope of the impact of wildfires on human health and well-being.

Prior research has identified some of the physical and mental health impacts of wildfires. Studies have shown that harmful pollutants, such as Particulate Matter (PM), contained in the complex smoke created from wildfires, can cause respiratory, cardiovascular and cerebrovascular issues when inhaled [13,14]. These health issues, which can range from asthma to heart disease, disproportionally impact survivors over 65 and those with underlying chronic illnesses [15]. Wildfires also impact physical health outcomes by contaminating local drinking water. A 2020 study found that both the Camp Fire and the Tubbs Fire caused drinking water contamination for local residents that exceeded federal and state exposure limits for several different contaminants including Benzene [16], which is known to increase cancer risk and to contribute to hematopoietic toxicity in the body [17]. Adverse mental health issues, such as depression, uncertainty, anxiety and PTSD, have also been observed in survivors of wildfires, both in the short and medium-term [18–20]. One study, which looked at the psychological repercussions of an Australian bush fire, found that the psychiatric morbidity of victims was double that compared to Australian communities that were not impacted by a fire [21]. Research looking into the psychological impacts of disasters more broadly, such as hurricanes and floods, have reported an increase in PTSD, depression, anxiety [22], substance use and suicidality in people living in the disaster-impacted area, particularly for young adults, and low-income survivors [23].

Disasters are also known to have considerable impacts on social determinants of health, such as housing and employment. For those who lose homes in disasters such as hurricanes and floods, rebuilding efforts can often take years, putting disaster survivors at risk of housing instability or homelessness [24]. Disaster survivors from socially marginalized groups, including low-income residents and communities of color, are most at risk of experiencing long-term housing issues following disasters [25]. Existing research on wildfires’ impact on housing and employment have been almost exclusively quantitative, focusing, for example, on the numbers of homes burned, the rate of rebuilding [26], or the extent of job loss in wildfire-impacted communities [27]. Few studies have captured the social and economic impacts of wildfires on survivor’s lives, a gap this research seeks to address. Moreover, past studies have focused primarily on the immediate survivors of wildfires; namely, those who have lost homes and/or loved ones. In contrast, our study examines the “downstream” social and economic effects wildfires can place on communities located near fire zones and the effectiveness of response and recovery programs implemented by public and private entities.

A number of governmental and civil society organizations provide health, social, and economic resources during the response and/or recovery phases. These phases refer to the period of time beginning from start of the disaster and lasting until the surrounding community has recovered from all of its impacts [28]. The Federal Emergency Management Agency (FEMA), the largest government-funded disaster agency, provided $347 million in grants to fire survivors in just two months following the Camp and Woolsey wildfires in November 2018 [29]. Humanitarian organizations, such as the American Red Cross also play a role. After the 2018 California wildfire season, the Red Cross allocated $27 million in financial assistance directly to survivors and committed another $11 million towards their “Community Recovery Program,” which provides grants to non-profit organizations who are helping wildfire survivors directly [30]. Community Based Organizations (CBOs), including community foundations, nonprofits and philanthropic organizations, are another large source of financial support for those recovering from wildfires. For example, one CBO, The North Valley Community Foundation, allocated over $30 million worth of grants to Camp Fire recovery efforts, providing both emergency assistance to survivors in the immediate aftermath of the fire, and long-term aid to the community [31].

Private and public recovery organizations are cumulatively spending billions of dollars providing relief to wildfires survivors. Yet, beyond occasional media coverage, there has been little investigation of the perceived impact and effectiveness of these recovery efforts among those who coordinate and/or use these recovery efforts. Some news articles have reported a lack of sufficient resources, especially for low-income residents [32,33], but in-depth research exploring this issue is lacking. This study aims to explore, through qualitative interviews with health and social service workers and volunteers, the health and social problems most prevalent among the wildfire survivors they have served in their roles, as well as their perceptions of the effectiveness of recovery programs designed to mitigate these impacts.

## Methods

### Research design

We developed a semi-structured interview guide and interviewed 21 health and social service workers about the experiences, perspectives, and challenges they have encountered assisting survivors of wildfires. Our research focused on the experiences of wildfire response and recovery personnel rather than fire survivors themselves because we wanted to understand the gaps in wildfire recovery resources from a systems level, and so gathered information from those who are working with these systems on a day-to-day basis.

### Participant selection

Study participants were recruited through purposive and snowball sampling. This approach enabled us to reach a wide variety of professionals, including physicians, nurses, public health workers, social workers, and nonprofit and philanthropic workers, who provided services during and following a wildfire. Any service worker or volunteer over the age of 18 who was directly involved in wildfire recovery and/or response efforts in California within the past four years was eligible for the study. We chose to analyze the results from Northern and Southern California fires jointly because the health and social issues that these fires create, as well as the pool of support resources available to survivors, are similar regardless of geographical location in the state.

Participants were recruited via local health and social service worker listserves, and through direct contacts with county agencies and nonprofit and philanthropic organizations. We also attended community meetings in fire-affected areas to recruit local response workers and asked participants to share the study information with their networks. Our participants do not represent every employee and volunteer position across each response and recovery effort, but rather, are a sampling from many of the services that fire survivors use to recover, both in the short and long term.

### Data collection and analysis

We utilized a semi-structured interview guide which was administered during in-person and virtual interviews. Consent was obtained orally at the start of each interview. Participants were asked to describe their roles and responsibilities during the wildfire response and/or recovery phase and were prompted to discuss three different areas: 1) the social issues that result from wildfires, 2) the health issues that result from wildfires and 3) response and recovery resources and their effectiveness. Data collection was terminated once interviewees were no longer presenting major additional concepts and thematic saturation was determined. Participants were interviewed between August 2019 and December 2019, one to two years after their respective fires occurred (see S1 File).

Interviews were stored and transcribed using a secure, encrypted online software. We manually extracted participants’ demographic data including profession, gender, and which fires they responded to. We used the software application, Dedoose, to facilitate qualitative analysis. Analysis involved three different steps: (1) categorizing concepts and ideas into broad domains and developed *a priori*; (2) using an inductive approach to continue to develop supplementary themes and deepen and subcategorize the pre-chosen domains; and (3) understanding nuances and differences in the responses among participants. Three a *priori* domains, health issues (both physical and psychological,) social issues, and the role of response and recovery resources, were explored through targeted questions. These three domains were initially selected based on our conceptual framework of the overarching themes that disaster-impacted communities encounter. As we coded our data, our team inductively subcategorized interview responses to deepen these themes and develop subdomains that were not initially established.

To ensure reliability, all coders were trained on the *a priori* codes. Coders then practiced assigning codes on sample data and discussed questions about coding with the rest of the study team. All new subthemes were formalized by at least two coders, who then trained the rest of the coding team on how to properly code data into these new domains. Interviews were coded by two different study personnel to ensure accuracy. Any discrepancies in coding were resolved via consensus with the coding team.

### Ethical approval

Ethical approval for this study was granted by the Human Research Protection Program at the University of California, Berkeley (Protocol ID# 2019-02-11800).

## Results

**Fig 1:**
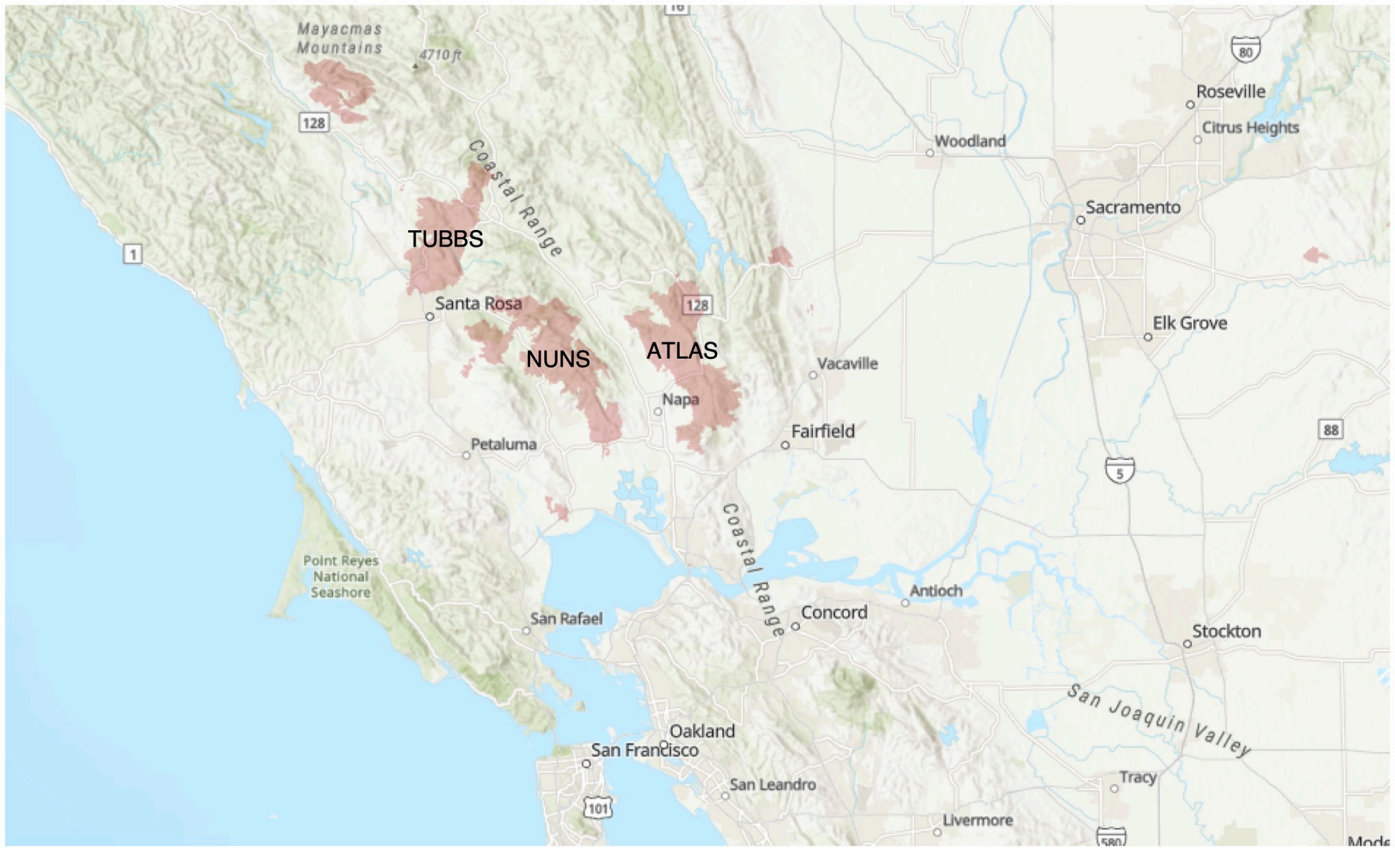
2017 North Bay Fires Burn Area (Tubbs, Atlas & Nuns).

**Fig 2:**
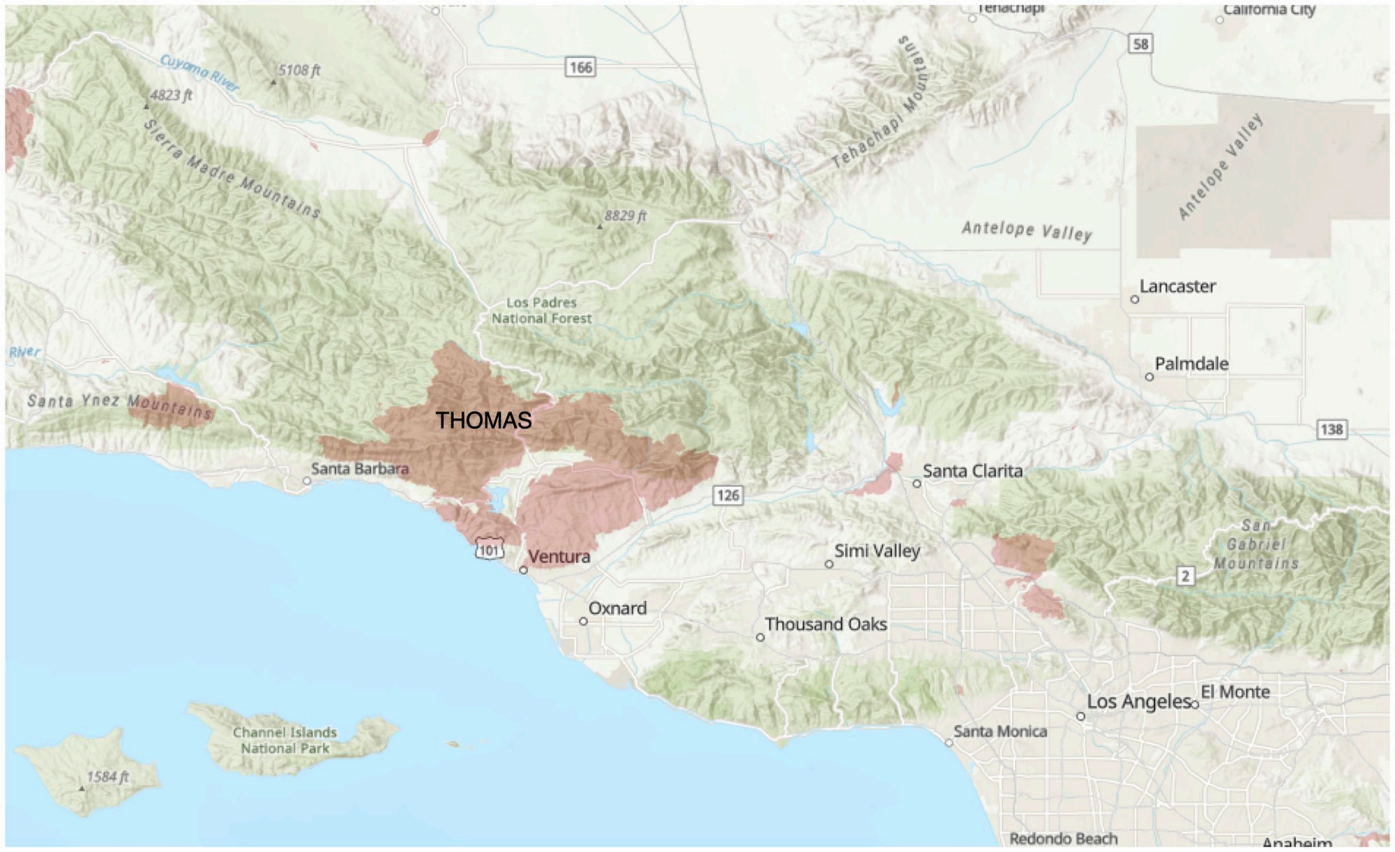
2017 Thomas Fire Burn Area.

**Fig 3:**
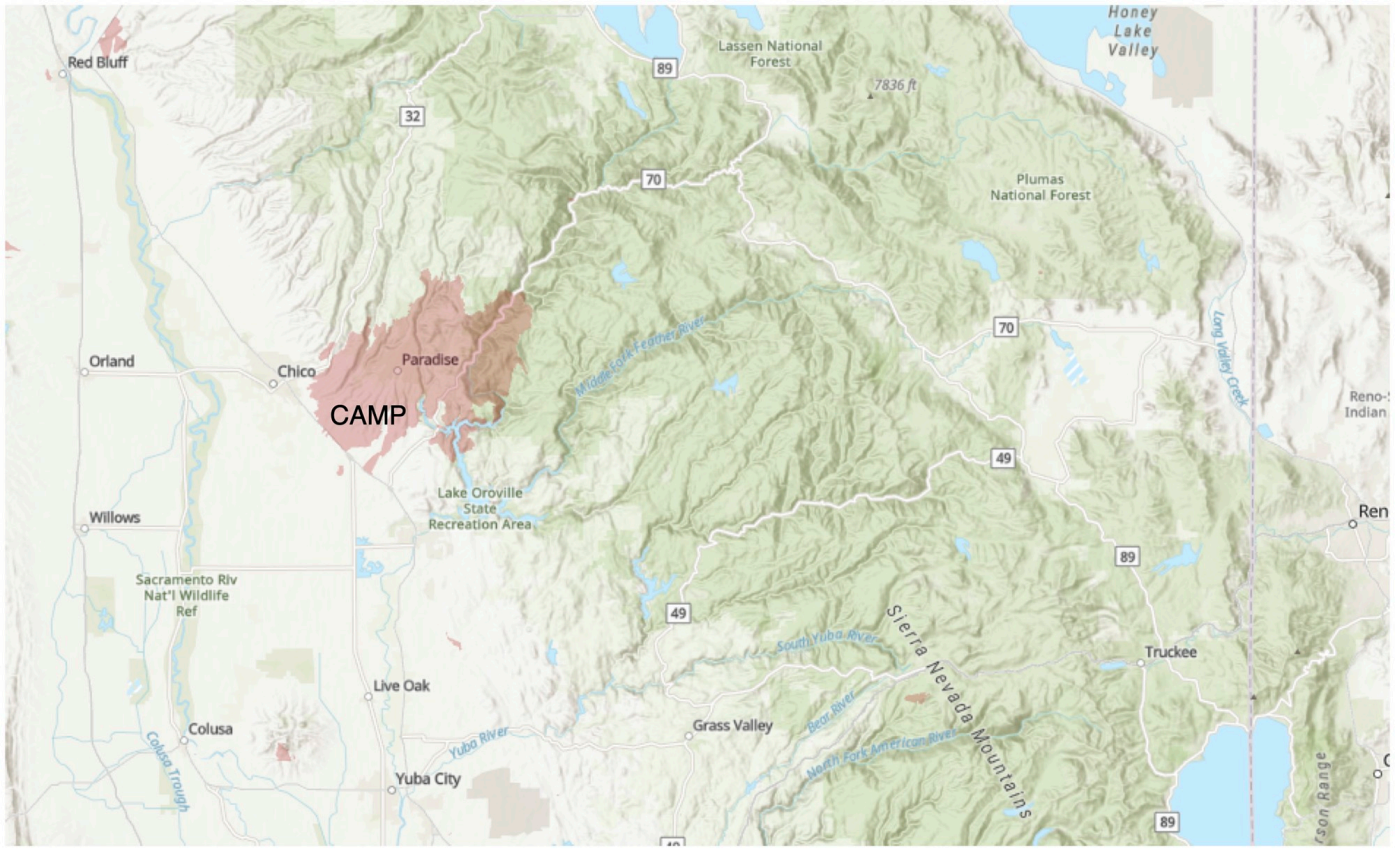
2018 Camp Fire Burn Area.

**Fig 4:**
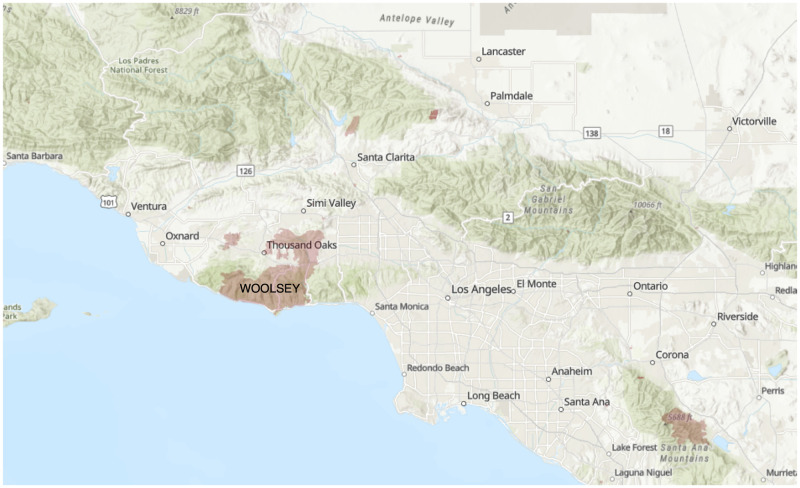
2018 Woolsey Fire Burn Area [35].

Forty-three community stakeholders initially responded and twenty-one agreed to be interviewed. Interviews lasted between 30 minutes and 75 minutes. Twenty interviews took place using Zoom audio [34], and one interview took place in-person. Nineteen interviews were audio recorded and two were not upon the participant’s request. Participants included eleven men and ten women; 20 participants held positions in the following fields: emergency medicine (8), philanthropy/nonprofit management (5), county health (4) and behavioral health (3). Lastly, one participant was a volunteer in shelter coordination. Most of the participants interviewed were not only professionals in the service sector, they were also members of the communities affected by the wildfires. We present the following analysis based on participant responses regarding their systems-level perspectives on the impacts of the fires, as well as their own personal experiences as service workers. Participants responded to four different fires in California in 2017 and 2018 (see Table 2). The following maps show the locations of the fires discussed in this paper: Figs 1–4.

**Table 2:** California wildfires that participants responded to.

Fire	County	Participants Interviewed
2017 North Bay (Tubbs, Atlas & Nuns)	Napa, Lake, Mendocino & Solano	10
2018 Camp Fire	Butte	6
2017 Thomas Fire	Santa Barbara & Ventura	2
2018 Woolsey Fire	Los Angeles & Ventura	1
Two or more fires		2

The following analysis captures three facets of the participant’s experiences during and after the wildfire: (1) social issues resulting from fires; (2) health issues resulting from fires; 3) response and recovery resources and their effectiveness. We sub-categorized the most commonly discussed social issues into: (1) housing, (2) employment. Health issues were sub-categorized into (1) mental/emotional health and (2) accessing health services. Response and recovery resources were sub-categorized into: (1) community support, (2) county agencies, (3) FEMA, (4) insurance companies and (5) philanthropy/non-profit.

### Social issues resulting from wildfires

#### Housing

According to the study participants, one of the greatest challenges that survivors faced following the wildfire was accessing safe and secure shelter. Participants described survivors undergoing an extended period of time when they moved around between different rentals, hotels or shelters. Study participants noted that the cumulative stress during this period of housing instability created or exacerbated physical health issues. “There have been so many people who’ve died post-fire and I think some of that has to do with the complications from being moved from a stable place,” noted a case manager who works at a nonprofit organization. A social worker echoed this relationship between increased mortality and housing insecurity:

“*I’ve had people tell me*, *‘Oh*, *my Dad wasn’t in a good place before the fire*. *And then we moved him and he was in a shelter and then he was in this new place and he died of a heart attack*. *And I think it was just all the stress of constantly not knowing where you were sleeping each night*, *where your meals are coming from*”.

The impact of wildfires on housing security and health is apparent for those whose homes were lost or damaged by the fire. Less obvious, however, is the ripple impact these wildfires had on the greater housing market. A county health official spoke about this phenomenon in Chico, the closest city to the town of Paradise which was heavily affected by the Camp Fire:

“*Almost overnight Chico became the number one housing market in America because houses that were sitting on the market or were not selling were selling now for $100*,*000 over asking price with bidding wars happening*. *And so our housing market went through the roof and our rental market just evaporated*. *We had no rentals*”.

A social worker who responded to the North Bay fires noted that the ensuing housing scarcity disproportionally impacted low-income residents. As local rents increased, safe and secure housing became increasingly difficult for community members already in financially precarious situations. According to the social worker, “Even people who were not directly impacted by the fires lost their homes because there is less housing available…So now people are in the streets because there isn’t anywhere for them to go. That’s been a huge challenge in the community”.

Residents who were affected by this ripple impact on the local housing market, moreover, did not have the access to recovery resources that were available to direct fire survivors. “Plenty of folks that I know have had homes sold out from under them,” a non-profit program manager said. “[S] ome of the stuff that kind of gets missed in all of the fire survivors stuff is that for those folks that were [in Chico] and got displaced by fire survivors, there is nothing for them”.

#### Employment

Wildfires presented serious consequences for the employment status of many local residents. Given the number of structures that burn in wildfires, many places of employment became damaged or destroyed, putting residents out of work. A county health official in Northern California noted how this phenomenon impacted one of the area’s prominent industries: “If a winery burned down or was damaged, that meant unemployment for people. So that was another impact”.

For some maintenance workers, residencies are their places of employment, so the loss of homes also lead to unemployment. A physician put it this way:

*The area where I’m living now*…*has a very high percentage of*…*doctors and lawyers and executives…And the executives employed a lot of…house cleaners [and other maintenance workers]… So a lot of gardeners lost their employment*. *I lost my home*, *so I’m not using my gardeners*… *So those people lost their sources of income*. *You know*, *so there’s that ripple effect through the community*”.

Workers who received hourly pay were also more likely to suffer unemployment as a result of the fires. While some salaried workers continued receiving paychecks while their workplace was closed down, many hourly workers stop getting paid altogether. “If clinics are closed,” a physician and hospital administrator said, “that introduces a whole other set of issues. You have staff that is paid hourly, their clinic closes, and they can’t work for a certain amount of weeks or months”.

Unemployment rates, created by the wildfire’s destruction, directly contributed to heath complications. Previous studies have shown that unemployment, and the subsequent economic instability it creates, can increase anxiety, depression, and certain types of cancer [36]. One participant in our study said that the loss of employment also resulted in an increase in housing insecurity in communities near fire zones. When survivors try to relocate to new homes, they often have to prove financial stability to landlords, which creates barriers to housing access. As this participant noted, “The other big issue is that a lot of people have lost jobs…And so now they’re having to show that they have good credit that they can make two and a half times the rent…most of our families don’t…and they’re having a hard time getting into housing for those two reasons”.

### Health issues resulting from wildfires

#### Mental and emotional health

Previous research has established that wildfires create significant impacts on the mental and emotional health of survivors, including increased anxiety, depression or PTSD [19,20]. Many participants in our study spoke about the prevalence and delayed onset of these sequalae. A county health official said that while survivors often focused on more immediate physical or material concerns, such as housing, mental and emotional issues were often not identified until months or years after the fire:

“*[Six] months in*, *people started [having] headaches and stomach aches*. *People [were] in high stress mode hitting a breaking point*. *You get people at the 6-month mark starting to seek health services for the first time*. *Then a year later you’re in actual grief*, *anxiety*, *anger going through the grief cycle*, *numb and disconnected*. *Then that can lead into depression*. *Going past that you start to see PTSD presentation*, *about 1*.*5 years later*”.

A physician who, at the time of interview, was already two years removed from the fire, also commented on how psychological issues in the community surface later:

“*Very few people come in and say*, *‘I have had anxiety since the fire*.*’ But a lot of people come in with anxiety or depression and when you get to talking to them*, *their lives were disrupted by the fire*. *Either they’ve lost their house or home or job or some other stressors have changed*”.

Study informants also reported wildfire survivors experiencing re-traumatization each year during wildfire season. A county health official noticed this phenomenon in his community:

“*So the disaster ends*, *but the community trauma continues*. *You’re going to have increased PTSD*, *you’re going to have greater anxiety in the community*. *Every time we’ve had a smoky critical incident of any kind our phone lines light up and people get really apprehensive*. *I think that is going to be a common experience*”.

Another troubling effect of ongoing trauma was its correlation to interpersonal violence within the community. Past studies have found that areas affected by disasters, such as earthquakes, floods or hurricanes, often experience higher rates of interpersonal violence including sexual abuse and child abuse [37]. Participants in our study reported similar trends happening in their communities. A psychologist noted: “There’s been upticks in domestic violence and child abuse reports, ostensibly from the added stress of not having a sustainable housing or employment troubles.” Similarly, a nonprofit program manager said: “There’s a lot of abuse. There’s been more…drug abuse, physical abuse…[and] more elder abuse”.

#### Accessing medical care

Accessing healthcare and addressing basic healthcare needs became a challenge for wildfire survivors, beginning from the moment the fire hit and lasting for years afterwards. During the immediate response phase, physical damage to pharmacies, as well as local road closures, meant that residents had difficulty accessing prescription drugs and other medical supplies. As a result, the few pharmacies that were open and accessible had trouble meeting the increased demand. One physician noted:

“*This is a nice little corner CVS [in our town]*, *but the pharmacy would quickly be depleted*. *And so how many [pills] are you going to prescribe*? *I’ll write a prescription for your diabetic medications*, *but I’m only going to prescribe a quantity of six pills because they can’t sell more than that*”.

Accessing healthcare not only became a problem for those who fled the wildfire, it also rippled throughout communities, particularly if doctor’s offices were damaged or destroyed. As a physician explained:

“*After the wildfire a lot of people lost their continuing care*. *They didn’t have a primary care doctor anymore…*. *I had one interesting case of a woman who had been misdiagnosed as having depression and anxiety from the wildfire for eight or nine months*, *but she actually had a malignant brain tumor*. *So*, *you can also get a lot of misdiagnoses*…”.

This disruption of primary care during and after wildfires also places an extra burden on hospitals and emergency rooms, as they often are the only operating medical facilities. According to a physician and hospital administrator we interviewed:

“*[L]ots of doctor offices weren’t even open for quite some time*. *So even if someone lost their medicine…*.*going to the ER was kind of one of the main places where you could actually go and see a doctor and get your medication*".

Wildfires often decrease the number of available health care workers who may themselves be impacted and unable to work. One participant, a physician, noted that over 210 physicians and health workers in his county of more than 180,000 residents lost their homes and were understandably unable to work. A shortage of staffing in a local hospital has the potential to put the health of the entire fire-impacted area at risk. A hospital administrator spoke about this issue occurring within a high-volume health care facility immediately after a wildfire:

“*What was fascinating that week was that all of the sudden*, *someone important was missing*. *They were there beside us helping and then all of the sudden it was like “Where did he go*?*” And it would turn out that he went or she went to go fight the fire that was closing in around their house*. *They went to defend their house*”.

Even when there was no wildfire, access to medical care could become problematic. A few participants spoke about the adverse impact the 2019 PG&E rolling blackouts, which were implemented to mitigate wildfire ignition, had on their communities. The blackouts, as a county health official noted:

“disproportionately affected…elderly people living by themselves that are socially isolated and…fragile. They might be on oxygen and during a power outage, the oxygen compressor doesn’t work….And what happens is sometimes they’re going to run out and we can’t get any more oxygen to them”.

### Response and recovery resources and their effectiveness

Wildfires can create significant health issues for communities in the present and well into the future. Meeting those health needs requires an abundance of resources. We asked study participants to speak about their perceptions of the effectiveness, accessibility and sustainability of available resources in their communities.

#### Community support

Immediately following the 2017 and 2018 wildfires, surrounding communities became one of the largest sources of financial and emotional support for survivors. Friends, relatives and neighbors pulled together to provide food, clothing, shelter, and other essential resources to survivors. Social media became a tool for organizing donations and showing support and solidarity with survivors. The Facebook group, *Camp Fire Butte County Resource Group*, for example, formed on the same day the fire broke out and became a central place where survivors could access information about resources, such as shelter, food, and basic medical care [38]. A physician spoke about the outpouring of community support following the fire: “People were saying ‘ I have a couch someone can sleep on. I have a bedroom…and I have an Airbnb’….the community pulled together big time. It was a rallying point for people”.

However, over time, the outpouring of emotional and financial support from the community started to dwindle. This decrease in community support, as a county health official explained, occurred even as survivors continued to struggle to meet their basic needs:

“*People got a lot of support initially… there was a lot of community outreach and a lot of people willing to take people into their houses*. *But after a couple months*, *people’s nerves were frayed a little bit and they weren’t so welcoming*…”.

Private donations became smaller and less frequent, and neighborly offers of shelter, money and other resources were less forthcoming. Another county health official put it this way:

“*People who originally put the welcome mat out to these people and were very sympathetic got to a point where they said*, *‘you need to deal with it*. *You need to move on*. *I’m tired of hearing about your trauma or your fire*…‴.

#### County agencies

County health agencies are responsible for a wide range of local health and safety measures, and following wildfires, often became the focal point for essential services for survivors.

Behavioral health departments within county agencies assumed an especially important role in promoting the physical and emotional wellness of survivors for several reasons. First, they were located within the impacted community, and their staff were already familiar with existing health resources and services. As such, they were well positioned to jump in and help coordinate response efforts. Still, there were challenges. One county behavioral health staff member spoke about the issues their staff faced in making sure that pre-existing clients were not falling through the cracks:

“*We had so many people displaced that our first step was to try and find them and make sure they were okay*. *So the big shift after the fire was mobilizing*, *providing crisis services and finding our clients as well as…providing services to people who weren’t really our clients pre-fire*”.

However, study participants noted that some county agencies that responded to the needs of fire survivors were often overwhelmed and unable to provide overall health services in their counties. As a county health official explained:

“*A lot of those services that we would normally be doing for the first couple months after the fire took a backseat to crisis response… We did a lot of services that were not reimbursable*, *which had a significant fiscal impact on our services*”.

Another county worker elaborated that this “fiscal impact” detracted from other essential services the county typically offered:

“*You have less discretionary funding for things like housing at the time when the housing is impacted*. *You have less money for transportation*, *you have less money for emergency food assistance…*. *Pretty much all of your support systems take a hit*”.

Though some of the services provided by counties are eligible for reimbursement by state or federal dollars, a county health official pointed out that economic repercussions for counties can still occur:

“*There’s a huge economic impact on local governments*. *…[R]eimbursement from FEMA and Cal OES isn’t necessarily super-fast…Without that money*, *core services get impacted*. *So then the very things that are being used to mitigate health impact are curtailed at the time when you have people that need it*”.

Ironically, counties often bear the financial burden of strategies to *prevent* wildfires. In 2019, PG&E, the gas and electric company that serves most of Northern California, administered power outages across the region in an attempt to mitigate the risk of fire ignition from downed powerlines [39]. A county health official spoke about the power outage in their community and its financial ripple effects, noting that money spent for managing the power outages is inevitably taken away from funds previously allocated to other health services:

“*Local counties don’t get reimbursement money for that*. *If we have [a] wildfire*, *we get the federal management assistance grant to deal with the consequences of wildfire… But for public safety power shut off*, *it…comes from County general fund*. *So it takes away from us being able to provide regular services*”.

#### Perspectives on the Federal Emergency Management Agency (FEMA)

FEMA is the federal government-funded agency that is responsible for disaster mitigation and response. Following a disaster, states can apply for FEMA funding on behalf of the impacted county or counties. FEMA’s Individual Assistance Program (IA) provides several types of disaster assistance to impacted households, including crisis counseling, unemployment assistance, legal services, case management and housing assistance [40]. Though this list of assistance services seems thorough and comprehensive, many study participants spoke about the challenges that survivors faced in receiving assistance through FEMA. A county health official described how provisions for housing were often slow in arriving in the immediate post-fire phase: “The FEMA trailers that were supposed to be coming to [our] county took an extraordinarily long time to be approved, to actually, physically land.” This was a frustration many study participants expressed. Another county health official said, “The FEMA housing is a consistent problem. Every disaster you hear about ‘where’s the FEMA housing?’”

Even if survivors were able to secure FEMA housing, it was only a short-term solution. The FEMA housing unit program is designed to provide shelter to survivors for up to 18 months after the fire [41]. After that, survivors are expected to secure housing elsewhere. A nonprofit program manager expressed concern over what would happen to low-income survivors when the FEMA program ended: “That 400-unit FEMA [housing] village has a hard end in May…. They’re going to just take people out. I don’t think they’re going to do their best to find people places to land. It’s one precarious housing situation to another, to another and to another”.

Wildfire survivors who applied for other individual assistance programs, such as FEMA grants, also faced issues in the process. A community health worker noted how the maximum FEMA grant is not enough to help survivors who may have lost everything: “Government does not ride in on a white horse and save you as an individual. The most you can get from a FEMA grant… is $33,000”.

FEMA’s case management program, which is intended to help connect survivors to basic resources like food, housing and medical care, was also criticized for leaving behind many survivors in need. A nonprofit program manager put it this way:

“*FEMA has had people on the ground since day one*, *but I think they’re woefully understaffed with what they need to do*, *particularly when it comes to case management*. *[L]ast I heard the waiting list to get hooked up with a case manager…is like 1*,*800 people long*, *which is several weeks for people who need help right now*”.

A nonprofit case manager noted that FEMA’s response to wildfire disasters was lacking because FEMA case managers were typically brought in after the fire from other parts of the country and were therefore unfamiliar with the disaster area: “These people are not well equipped to be working on the ground here. They don’t know the community. They don’t know the people. And they don’t know the resources”.

#### Insurance

Homeowners and renters insurance is so central to survivors’ recovery that, as of January 2019, Californians had filed $11.4 billion worth of insurance claims for their destroyed or damaged homes during the 2018 fire season, one of the highest claim seasons reported by insurance companies on record [42].

While one study participant noted how helpful insurance companies can be immediately following the disaster, many others reported challenges to navigating the insurance system. For many survivors, attempting to claim insurance funding became an extremely stressful and arduous process. Study participants noted that many survivors experienced difficulties acquiring the total sum of money they thought they were eligible for. A nonprofit program manager who has helped survivors file insurance claims said:

“*For most people*, *the biggest shock is finding out that their financial lifeline*, *their insurance policy*, *the limits are way too low to put them back where they were*. *Two thirds of the people that lose their homes in wildfires find themselves severely under-insured*”.

Indeed, homeowner’s underinsurance is widespread, affecting about 60% of Americans [43]. Yet even if survivors are eligible for a higher insurance claim, they can experience significant logistical problems and delays while navigating this process. A nonprofit program manager explained why survivors ended up jumping through so many hoops:

*I mean*, *if you*’*re willing to take whatever money the insurance company offers you*, *then fine*, *make the best use of those funds that you can*. *But if you want to really max out your policy…and collect every dime…it takes work and it takes time*”.

Work and time are not the only pieces needed to gain the maximum amount of claims that survivors may be eligible for. A nonprofit program manager added that strong networks, computer literacy, and fluent English skills are qualities that significantly increase one’s chances of receiving a higher claim, revealing an unfair disadvantage for elderly people and English language learners as well as those struggling to work, care for dependents and manage other needs. The same participant added: “Isolated seniors do the worst. They get taken advantage of by insurance adjusters that perceive them to be trusting…and certainly anyone for whom English is a second language”.

#### Philanthropy

Several participants noted that the cumulative support from county and federal programs, insurance companies, and family and friends was not sufficient to adequately address survivors’ basic needs after a wildfire. Therefore, nonprofits and philanthropic organizations often became essential players in helping wildlife survivors and their communities recover. Speaking about the advantages of receiving recovery funds through philanthropic organizations, a county health official noted: "They’re fast and they don’t have to go through a lot of government bureaucracy and red tape…” A nonprofit program manager working in the philanthropic world noted how nonprofit services filled in the “donut hole of things that don’t get funded by either insurance or the government…Whether that’s keeping a preschool open or helping people get propane so they can keep warm in the winter”.

Several participants noted that another huge advantage that philanthropic and non-profit recovery services have over federal and state recovery services is that they are usually available to residents, such as undocumented immigrants, who are either ineligible or otherwise unable to access other systems of recovery. One country health official put it this way: “Health equity during disasters worsen because you have undocumented immigrants that are afraid to get aid. Because of fear of ICE [Immigration and Customs Enforcement] or fear of deportation. So the CBOs [Community-Based Organizations] [are] a trusted resource for those more marginalized populations”.

However, the scale of destruction that wildfires create is challenging for any private organization to address, regardless of how well they fundraise. Reflecting on the amount of money that his organization raised following a fire, a nonprofit program manager said: “[Tens of] million[s] is a lot of money, but the disaster was estimated somewhere between $15 and $20 billion. Billion, with a B. So philanthropy isn’t going to rebuild {de-identified town} or fix all the problems”.

Even more alarming, many participants, including some who work for philanthropic organizations, expressed concern about the long-term sustainability of these funds as wildfires in California continue to worsen. The “donor fatigue” phenomenon, though yet to be fully substantiated by research, may be starting to develop in communities that see destructive wildfires year after year. A nonprofit manager noted:

“*I have heard some concern from some of the other organizations that have done philanthropy and are involved in homebuilding and*…*as there had been more fires that are popping up*, *they’re starting to see what they’re calling ‘fire fatigue’ on the side of donors*… *People that have been raising a lot of these funds for disasters for a long time are saying it’s having an impact*. *It’s another fire*, *you know*”.

## Discussion

The findings of this study highlight some of the community-level, immediate and downstream health and social impacts of wildfires in California. The study also provides an in-depth look at the perceived effectiveness of some of the recovery resources that fire survivors use to regain emotional, physical and financial wellness following a fire. While wildfires significantly impact the physical health of survivors, the social, emotional and economic impacts—and their ripple effects on the community—are critically important to address. Our study draws attention to the importance of examining the intrinsic connection between the health and social outcomes of wildfires, and the availability of recovery resources. The results of this study highlight how essential comprehensive and long-term recovery resources are in survivors’ healing trajectories. Moreover, although this study was conducted before the COVID-19 pandemic, it highlights the vulnerabilities that fire-affected communities and systems face if they need to simultaneously manage an additional disaster, such as a pandemic, during the fire recovery process.

Many of the health and social issues that participants spoke about in our study—housing, employment, mental health, and access to healthcare—echo previous research about how disasters impact these health domains. Like previous research on housing and disasters, our study found that those who lose homes in disasters face long-term rebuilding and/or resettling trajectories, particularly if they are low-income [24,44]. Our research captured qualitatively some of the ways that housing issues following a wildfire have direct implications on health outcomes, with participants telling stories about both morbidity and mortality deriving from issues of housing insecurity, noting that low-income wildfire survivors were most at risk. On a community level, our study expands our understanding of the scale of housing issues on surrounding areas. Where previous studies looking at housing and wildfires have focused on direct impacts, (e.g., homes that are damaged or destroyed in the wildfire), [26] our results point to a larger, ripple effect on housing, creating housing shortages and contributing to rent increases in the surrounding communities.

Our study also supports prior research that connects a rise in local unemployment rates to the disaster, both in the short and in the long term. Prior research has largely captured the impact of disasters quantitatively through examining unemployment rates in the months and years following the event [27]. Our study captured some of the nuanced individual experiences of employment loss following wildfires, including its role in contributing to further housing insecurity, and thus impact on health. The study also underscores that the loss of housing has a direct impact on employment, as one home’s destruction will inevitably lead to the unemployment of everyone who might work in that home.

Our findings on wildfire survivor’s mental and emotional health aligned with prior research that documented higher rates of anxiety, depression and trauma in disaster-impacted areas [18,20]. The health and social service workers that we interviewed emphasized however, that those who experience adverse mental health issues following wildfire disasters often do not begin to recognize and address these mental health issues until months or years after the fire. Moreover, the complex interaction between experiencing fires, caring for those in need, and recovering from social and economic impacts, may exacerbate psychological harm. Our participants also described how survivors’ fire trauma and anxiety can become reactivated each year when the wildfire season resumes. These findings point to the importance of securing sustainable and long-term mental health services for those impacted by the fires, not only to process the initial shock of the disaster, but to address long-lasting and reoccurring trauma symptoms.

Prior studies examining healthcare access following wildfires have primarily focused on the uptick of emergency room visits [14,45]. Our study provides insights into the capacity of healthcare *workers* as well as healthcare facilities following a wildfire. The participants we interviewed who worked in hospitals and responded to the immediate health needs of survivors reported significant worker capacity issues in the response phase. Because the fire has the potential to devastate the entire community, many healthcare providers also become survivors of the wildfires themselves, limiting their capacity to perform at work and thus compromising the staff capacity at the hospital. Hospital staffing capacity issues that arise during disasters are especially important to consider as we navigate the COVID-19 pandemic. Responding to wildfire crises alone can put the local medical system in a precarious position. Adding yet another medical disaster, such as the COVID-19 pandemic, may push certain communities well beyond their breaking point, and further limit already scarce resources, ultimately leading to an increase in preventable deaths in the local community.

Crucially, our interviews point to a crisis in recovery resources that will only get worse in coming years as climate change intensifies. While speaking about the individual and community-wide health concerns they addressed in their roles, participants of the study, who occupied a wide range of health and social service professions, expressed great concerns with both the comprehensiveness and sustainability of wildfire recovery programs. The support given by surrounding community members, though initially robust, is not long-lasting. FEMA provides a variety of different recovery programs that can help with housing, finances, case management and more, but study participants expressed disappointment with the quality of FEMA’s services and frustration with the short-term nature of its programs. Homeowners insurance, which many survivors hope will be, as one participant noted, “a financial lifeline,” in fact leaves many residents without the resources needed to rebuild their homes. Local county agencies currently assume primary responsibility for responding to the immediate issues following a wildfire, but response efforts are costly, and though county agencies eventually get reimbursed by the federal government for their expenses, these agencies often nevertheless end up taking a financial hit. The downstream impacts of this financial hit can be dramatic—county agencies must shift funds around, cutting budgets for other essential community health programs.

Given the gaps in these various resources, philanthropic agencies have been providing a large amount of necessary aid to wildfire survivors, particularly those without access to other forms of aid, such as undocumented migrants. Indeed, aid funneled through philanthropy is often the most crucial recovery recourse, providing essential health and social services not only in the immediate aftermath, but well into the long-term recovery phase. The North Valley Community Foundation in Butte County, for example, co-sponsors *The Butte Strong Fund*, whose purpose is to provide survivors with long-term aid in the form of housing, healthcare, business recovery, community development and more [31]. Given that aid provided by government is short-term, philanthropic organizations can become the *only* source of recovery aid available to address the long-lasting impacts of wildfire disasters.

Participant’s comments, therefore, about the worsening wildfire seasons contributing to “fire fatigue” on behalf of philanthropic donors, are greatly concerning. Interviewees noted that, already, philanthropic organizations were concerned about a downturn in dollars raised after recent wildfires, suggesting that donors might be growing tired of putting money into wildfire relief efforts year after year. Though the “fire fatigue” phenomenon should be further explored in research, these comments underscore the urgency to examine the sustainability of our current recovery resources and explore whether we are currently relying too much on philanthropy efforts to meet the health needs of wildfire survivors.

Given the scientific evidence indicating that wildfires will continue to worsen in California as climate change progresses [12], local, state, and federal policymakers must seriously consider the weaknesses of our current health, social and economic recovery resources. They must also implement innovative structural changes to meet the needs of wildfire survivors and their communities.

First, more federal and state resources must be dedicated to local, community-based wildfire recovery programs. FEMA has extensive resources, but these appear to be inconsistently deployed. And while philanthropy currently plays a large part in supporting recovery efforts, the ‘fire fatigue’ phenomena suggests that a more permanent and consistent means of financing local, community-level recovery programs must be implemented and sustained. In doing so, an increase in federal funds must be allocated to disaster recovery services. These extra funds would be best delivered through local and state government programs, with private philanthropy serving as a supplemental source of support.

Second, since the health and social impacts of wildfires can last for years after the final flames flicker out, recovery programs must be developed to attend to the long-term needs of survivors and their communities. These recovery efforts should include support for housing, employment, and access to psychosocial services. Such programs should be mindful of the special needs of vulnerable groups, such as the elderly, individuals with disabilities, undocumented migrants, low-income residents, as well as community members with pre-existing diseases or medical conditions. As policymakers develop these programs, they must also anticipate and plan for the “unknown,” such as pandemics and other health emergencies that may already be overburdening existing health facilities.

And, finally, recovery programs must anticipate and respond to the “ripple effect” that wildfires have on local and county services in surrounding communities. Our study highlights the ways in which health issues can ripple into the community, putting the health and safety of those not initially impacted by the fire, at risk. Dramatic changes to the local housing market can price residents out of their home and communities; families receiving social services through their county agencies may feel the impact of budget cuts on these essential health services; and the limited capacity of hospital workers following the fire could impact the quality of emergency care for the entire surrounding community.

## Limitations

Limitations of our study include a relatively small sample size. Our 21 participants responded to four different wildfires, each different in magnitude and impact, which may not have given us a comprehensive representation of all experiences related to the health impacts of wildfires and recovery efforts. Furthermore, our study centered the experiences of workers who chose to respond to our call for participation, possibly contributing to selection bias. Another limitation is that some participants were months or years removed from the experiences they were speaking about and it is possible that the events were no longer fresh in their minds, possibly contributing to recall bias. Additionally, member checking was not utilized in this research study which many have comprised the full accuracy of participants’ statements. This was an exploratory study into the health and social impacts of wildfires and much work is needed to continue to understand these impacts and assess and address the vulnerabilities in wildfires response services.

## Conclusion

This study provides an overview of some of the most salient health and social issues that wildfire survivors face following the disaster. Beyond the immediate physical health problems, such as the cardiovascular issues that have been well documented by past research, this study presents a more nuanced look at the health and social issues that survivors experience both in the short and long term. The study centers the voices of health and social service workers who are implementing response and recovery efforts for wildfire survivors both in the immediate response phase of the disaster, and for years following. Their perspectives highlight the cyclical relationship between health and social outcomes, including housing, employment, mental health and access to health services, and the accessibility of recovery resources following a wildfire. Their stories underscore that helping survivors recover emotionally, financially and physically, requires comprehensive and accessible response and recovery resources. Without access to these services, survivors, particularly if they were limited resourced to begin with, can experience severe and long-lasting health and social problems. Our study is exploratory, and it is our hope that our preliminary findings will be tested in larger qualitative and quantitative studies with the aim of developing more effective programs to aid survivors of wildfires and their communities. Given the increased severity and pervasiveness of wildfires in the U.S. and abroad, we must understand how to better meet the needs of those whose health and well-being are most impacted by these disasters.

## Supporting information

S1 File (DOCX)
