# Supplementary material for: Health and social impacts of California wildfires and the deficiencies in current recovery resources: An exploratory qualitative study of systems-level issues
Source: PLoS One. 2021 Mar 26;16(3):e0248617. doi: 10.1371/journal.pone.0248617 (PMC7997008; doi:10.1371/journal.pone.0248617)
Supplement: S1 File — (DOCX) [file pone.0248617.s001.docx]

**Interview guide**

1. *Tell me a little bit about your role and how and when you came to work for [insert organization.]*
2. *Can you tell me about [insert organization?] What do its services look like?*
3. *Why is the work you do important for survivors of wildfires?*
4. *What were some of the immediate health issues you or your organization were working to address?*
5. *We are currently ___ years after the fire. What health issues, if any, is your organization still addressing?*
6. *Is there a particular demographic that [inset organization] serves? In other words, are there any trends that you notice in terms of who needs ongoing health services the most?*
7. *What is the plan for terminating support services, if there is one? How will you know when the services are no longer needed?*
8. *What do you think are the most serious issues experienced by survivors of wildfires overall?*
